# Detection of Hepatitis C virus RNA using a novel hybridization chain reaction method that competitively dampens cascade amplification

**DOI:** 10.1371/journal.pone.0268917

**Published:** 2023-03-10

**Authors:** Chen Zhang, Qingrong Qu, Yuming Yao, Xiaobo Fan, Guoqiu Wu

**Affiliations:** 1 Department of Diagnosis, Medical School, Southeast University, Nanjing, People’s Republic of China; 2 Center of Clinical Laboratory Medicine, Zhongda Hospital, Southeast University, Nanjing, People’s Republic of China; 3 Jiangsu Provincial Key Laboratory of Critical Care Medicine, Zhongda Hospital, Southeast University, Nanjing, People’s Republic of China; 4 Department of tuberculosis, Shanghai Pulmonary Hospital, School of Medicine, Tongji University, Shanghai, People’s Republic of China; University of Pennsylvania, UNITED STATES

## Abstract

The hybridization chain reaction (HCR) is widely used for biosensing. However, HCR does not provide the required sensitivity. In this study, we reported a method to improve the sensitivity of HCR by dampening the cascade amplification. First, we designed a biosensor based on HCR, and an initiator DNA was used to trigger the cascade amplification. Optimization of the reaction was then performed, and the results showed that the limit of detection (LOD) for the initiator DNA was about 2.5 nM. Second, we designed a series of inhibitory DNAs to dampen the HCR cascade amplification, and DNA dampeners (50 nM) were applied in the presence of the DNA initiator (50 nM). One of the DNA dampeners (D5) showed the best inhibitory efficiency of greater than 80%. This was further applied at concentrations ranging from 0 nM to 10 nM to prohibit the HCR amplification caused by a 2.5 nM initiator DNA (the limit of detection for this initiator DNA). The results showed that 0.156 nM of D5 could significantly inhibit the signal amplification (p<0.05). Additionally, the limit of detection for the dampener D5 was 16 times lower than that for the initiator DNA. Based on this detection method, we achieved a detection limit as low as 0.625 nM for HCV-RNAs. In summary, we developed a novel method with improved sensitivity to detect the target designed to prohibit the HCR cascade. Overall, this method could be used to qualitatively detect the presence of single-stranded DNA/RNA.

## Introduction

Hairpin DNA-mediated strand assembly (HDMSA) is an isothermal and enzyme-free amplification technique that is mainly divided into the hybridization chain reaction (HCR) and catalytic hairpin assembly (CHA) [1]. HCR and CHA were first described in 2004 [2] and 2008 [3], respectively. The hairpin DNA-mediated isothermal amplification (HDMIA) reaction can be conducted under isothermal conditions without enzymes and thermal cyclers. This procedure is considered superior to the PCR method in simplicity, reaction rate, and the equilibrium amount of captured targets [1,4]. A good signal-to-background ratio was achieved using HDMSA with the labeled hairpin probes, and the reported detection quality was superior to other traditional sensing schemes [4–9].

HDMSA coupling to various signal readout techniques, such as fluorescence [10–13], chemiluminescence [14,15], colorimetry [16], photoelectrochemistry [17–19], and electrochemistry [20–24], has now been widely applied for biosensing of DNAs/RNAs, proteins, organic compounds (such as ATP and antibiotics), ions and so on. Most of the reported detection limits of DNA/RNA for pure HDMSA (without signal magnifiers) were between the nM to pM range because of the strong background that accounted for the low fluorescent quenching efficiency [5,6,10]. Electrochemistry-based HDMSA seemed to be more sensitive, with an improved limit of detection (LOD) below the pM range [25,26]. HDMSA was usually coupled to a signal magnifier system based on enzymes or nanoparticles to further improve the sensitivity. Some of the enzyme-aided HDMSA strategies have proposed detection limits comparable to that achieved by PCR [27,28]. However, the engagement of enzymes greatly overrides the main advantage of HDMSA as an enzyme-free technique. The vulnerability of enzymes significantly limited the applications of enzyme amplified-HCR nucleic acid assays in complex biological samples and was also not convenient for transport and storage. Alternatively, both G-quadruplex/hemin and DNAzyme, which were dependent on the catalytic activity of the DNA/RNA itself, somehow overcame the drawbacks of protein-based enzymes and were successfully applied in many studies [29–32]. In addition to the enzymes, nanoparticles harboring complexes with fluorescent or enzymatic components are reported to be an effective strategy for amplifying the signal and significantly improving the LOD [33,34].

While most of the attention was drawn to designing a more sophisticated signal magnifier, a more robust and efficient hybridization chain reaction system, termed dendritic or branched HCR [35,36], was reported to achieve exponential growth, resulting in higher signal intensity, better resolution, and lower detection limits. However, no further research was carried out based on this method, which may have resulted from the poor controllability and leakage reaction.

Previous studies have highlighted the design of specific hairpins and sophisticated signal-enhancement strategies to improve assembly efficiency while avoiding target-independent assembly [1,8,9]. We have also reported on the application of HCR coupled with the lateral flow immunoassay for the rapid and sensitive detection of viruses with a LOD within the pM scale [12]. However, we found that the sensitivity was inadequate, and at least 24% of positive samples were beyond the LOD. The pure HCR/CHA was insufficient, with most reported LOD occurring at the nM scale. We, therefore, carried out this study to explore a new method to improve the LOD of a pure HCR system. This study focuses on the cascade amplification and report a new method to improve the LOD by manipulating the assembly cascade.

## Materials and methods

### Chemicals, reagents, and samples

The DNA oligonucleotides used in this work were synthesized and purified by Sangon Biotech. Co., Ltd. (Shanghai, China). Bovine serum was purchased from Sigma Aldrich (Shanghai, China). Hepatitis C virus RNAs (HCV-RNAs, National Institute of Metrology, Beijing, China) were obtained from ZhongDa Hospital. Unless stated elsewhere, all chemicals used in this study were analytical grade and obtained from Aladdin (Shanghai, China).

### Design of the hybridization chain reaction hairpins for hepatitis C virus (HCV) detection and inhibitory sequences for the HCR

The complete genome sequence of HCV was obtained from the NCBI database and analyzed to retrieve the conserved sequence for HCV detection. The conserved sequences were further analyzed by NCBI BLAST against the reference RNA sequences. Two sequences of 23 nt without secondary structure were chosen for HCR design in NUPACK [37].

### Preparation of the DNAs and HCV-RNAs

The designed sequences were sent to company for synthesis and purification. The hairpins were prepared by diluting each DNA with TAE/Mg^2+^ buffer (40 mM Tris-Acetate, 1 mM EDTA, and 12.5 mM magnesium acetate, at pH 8.0) to reach a final concentration of 1 μM, and annealed over a temperature gradient between 95°C and 25°C for 90 min. The prepared hairpins were preserved at 4°C for future application. Additionally, the initiators, dampeners, and HCV-RNAs were dissolved in bovine serum and stored at 4°C for future application.

### Theoretical calculation

For the theoretical calculation and modeling, the free-energy changes of each reaction were estimated by the NUPACK software package [37]. The free energy (ΔG°) change for the hybridization reaction is calculated using the Van’t Hoff formula (Formula 1).

$$\Delta G^{0}=\Delta G_{reactants}-\Delta G_{products}=\Delta H^{0}-T\Delta S^{0}=RT\;\ln\;K_{eq}$$

where ΔH and ΔS are the changes in enthalpy and entropy of binding, respectively, T is the temperature (K), R is the universal gas constant (8.314 J/mol·K), and K represents the equilibrium constant.

### Native-polyacrylamide gel electrophoresis

DNA hybridization was conducted at 25°C for 1–2 h. Next, the reactions were examined by gel electrophoresis (120 V, 1h). TAE buffer was used as the electrophoresis buffer. Finally, the gels were visualized under UV light at 280 nm after being stained with 10 mg/mL ethidium bromide (EB) for 20 min.

### Fluorescence measurements

All fluorescence measurements were performed on a PCR amplifier (CFX-96, Bio-Red, Shanghai, China) or a spectrofluorometer (Fluoromax-4, Horiba Ltd., Shanghai, China). The H1 hairpins are labeled with both 6-carboxyfluorescein (FAM) fluorophore and BHQ quencher at two ends. The fluorophore was quenched by the adjacent quencher when the H1 was maintained as a hairpin. The hybridization of H1 with the initiators or other hairpins opens up the hairpins and releases the fluorescence [12].

### Optimization of reaction conditions for the HCR reaction

Three initiators of different lengths were designed for this study. First, the reactions containing 200 nM hairpins (Fam-H1-BHQ, H2, H3, and H4) and 200 nM random DNA in TAE buffer supplemented with different concentrations of the initiators were incubated at RT for 2 h, and the fluorescence intensities were recorded. The dynamics of the DNA hybridization were closely related to the component ratio, reaction time, and temperature [12]. To obtain the best limit of detection (LOD) for the DNA initiator, the reaction steps were optimized with these parameters. The reaction was first optimized similarly after supplementation with the initiator I1 under RT, and the fluorescence intensities were recorded over time. To optimize the reaction conditions with the best resolution, the molar ratio was optimized by supplementing different H2–4 concentrations ranging from 100 to 400 mM to the reactions containing 100 mM of Fam-H1-BHQ, while H2, H3, and H4 were added at an equal molar concentration. Furthermore, the LODs for initiator I1 were measured at different hairpin concentrations applied in a fixed molar ratio of 1:2 (H1:H2–4), and the Fam-H1-BHQ concentration was changed from 25 to 200 nM.

### Screening of DNAs inhibiting the HCR cascade amplification

**Fig 1:**
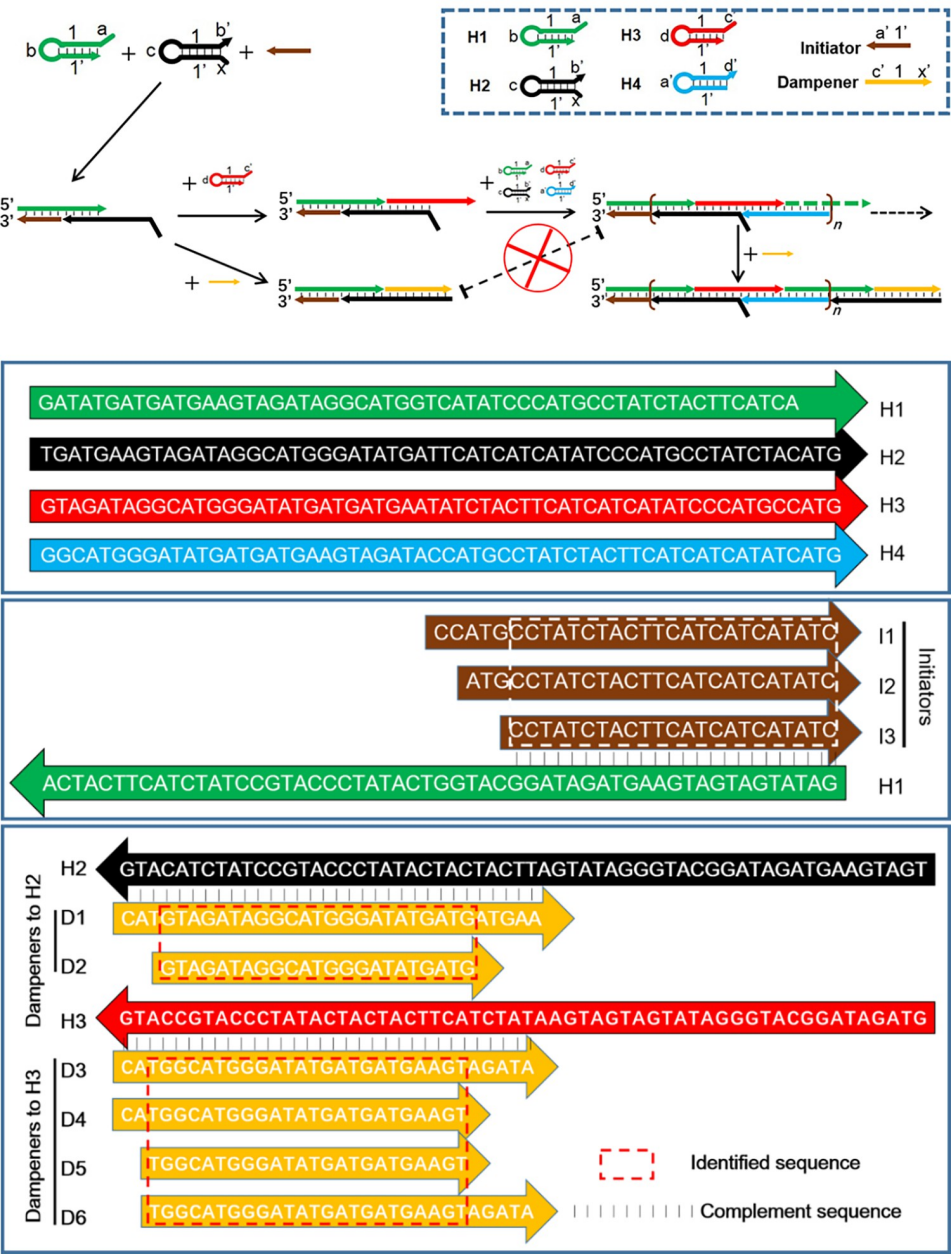
Principle and sequence design for this study. A) Schematic for the principle of the dampeners to prohibit the HCR hybridization cascade. B) The sequences used in this study.

Fig 1 shows that a series of DNAs termed “dampeners” complementary to the exposure region of H3 during the hybridization chain extension were designed to prohibit the cascade amplification. To screen for effective dampeners, dampeners at equal concentrations to the DNA initiator were added in each reaction at the optimized conditions. The inhibitory threshold was calculated, and the dampeners with >80% inhibition were sent for further selection. To demonstrate the specificity of this method, several non-target DNAs was designed and applied in the reaction (S1 Table).

In the second round, a series of concentrations of the DNA dampeners were added to the optimized reaction in the presence of the DNA initiator at a concentration equal to its LOD. The LOD for the dampeners was then calculated.

### HCV-RNAs detection

The dampener D5 was a 20-nt sequence originating from the HCV genome. To verify this method in HCV detection, we applied this method to detect HCV-RNAs. A series of concentrations of HCV-RNAs dissolved in bovine serum were prepared and measured using this method. Unrelated linear DNAs were used as blank controls.

### Data processing and statistics

The results were expressed as the mean ± standard deviation (SD). ANOVA test was performed to compare the differences between groups. Values of p<0.05 were regarded as statistically significant.

## Results and discussion

### Design of the sequence

The HCV genome sequence was retrieved from NCBI and analyzed with BLAST (https://blast.ncbi.nlm.nih.gov/Blast.cgi). A specific sequence was retrieved and used as the template for the design of the dampeners. Based on the dampener sequence, a hairpin-based HCR system consisting of four hairpins and two linear DNAs was designed with NUPACK [37,38]. Fig 1A shows that the DNA initiator bound the H1 toehold region and released the hidden region for H2 hybridization. H3 modified with both a fluorophore 6-Fam and a quencher BHQ-1 was used as the reporter beacon. The H3 hairpin was open when it hybridized in the complex and produced a detectable fluorescent signal. The dampener was designed to, for example, target the exposed region of H2 without leaving a sticky end for the downstream H3 hybridization. Like competitive enzyme-linked immunosorbent assay (ELISA), the dampener target competed with H3 to bind with the H2 toehold and thus, broke the cascade hybridization caused by the initiator. In the presence of a given concentration of the initiator, more dampeners caused more inhibition of the cascade hybridization caused by the initiator.

The hairpins were designed with a 6 nt toehold region, 12–18 nt stem, and 6 nt loop [38,39] to ensure a low background and a rapid hybridization rate. To select the most effective dampener, a series of DNA dampeners of different lengths were designed based on the template (Fig 1B).

### Energy simulation

S1 Fig demonstrates the predicted secondary structures of the DNA hairpins with Gibbs-free energy (ΔG) at different temperatures. The Gibbs-free energy (ΔG) of each hybridization reaction product at different reaction temperatures is shown in Table 1. The principle of this study was based on the theory that in competition with hairpin H4, the dampener could more effectively bind with the x-H1-H2-H3 (x = initiator-(H1-H2-H3-H4)_n_-) complex. The HCR reaction consisted of four hybridization steps and numerous reaction byproducts. It was difficult to calculate the equilibrium concentrations for each complex. However, the simulation results showed that in competition with H4, the incorporation of the dampener yielded a much bigger change in the Gibbs-free energy compared with extension by hybridizing H4 and/or H1. The linear dampeners that formed extra base pairs after hybridization were energetically favored because base paring released heat [40,41].

**Table 1:** The change in Gibbs-free energy for the different complex products.

Complex product	Production of Gibbs-free energy (Kcal/mol)
Ini-H1-H2-H3	-37.98
Ini-H1-H2-H3-H4	-55.06
Ini-H1-H2-H3-Damp	-72.45
Ini-H1-H2-H3-H4-H1	-69.1

### Optimization of the HCR reaction conditions

The results showed that the initiator I1 had the best ability to trigger the hybridization cascade and the greatest fluorescence intensity at all test concentrations (S2 Fig). As the longest linear initiator, I1 could form more base pairs after hybridizing with the hairpin H1, yielding a bigger free energy loss [41,42]. Therefore, a longer linear initiator was energetically more favorable for the hybridization chain reaction. The initiator I1 was therefore used for the following experiments [42].

The results of the fluorescence intensity over time indicated that the hybridization chain reaction was characterized by two stages (S3 Fig). The intensities increased in the first several minutes and then slowly plateaued within 1 h. To save time, the reaction was optimized to occur for 30 min.

**Fig 2:**
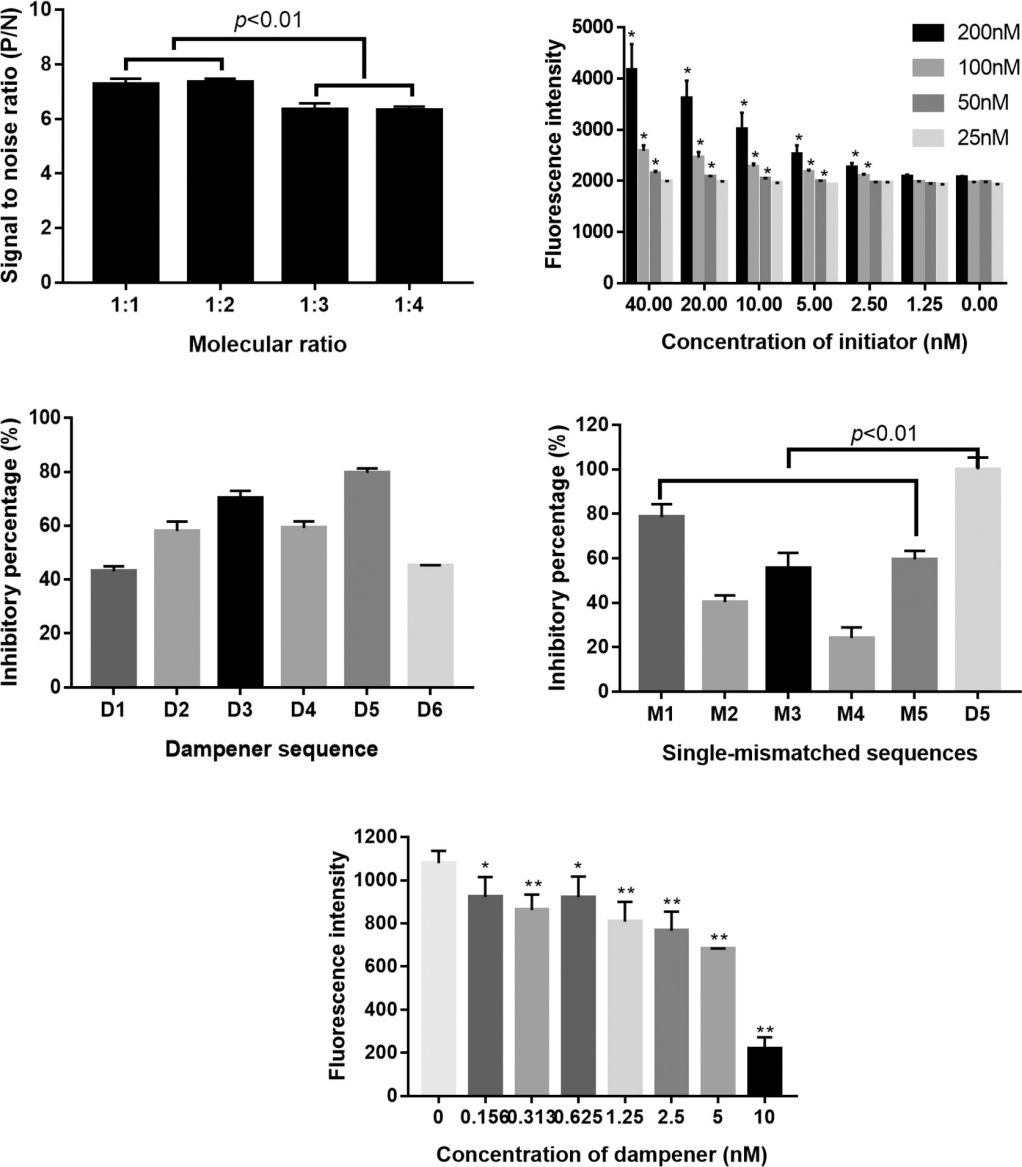
Optimization of the HCR with different parameters. A) Optimization of the molar ratios of H1:H2–4. B) Determining the LOD of initiator I1 at different concentrations of hairpins. * p<0.05 vs control added with 0 nM initiator. C) Inhibitory efficiency of different dampeners. D) E) The inhibitory effect of dampener D5 in the presence of I1 (2.5 nM). * *p*<0.05 or **p<0.01 vs control added with 0 nM dampener. Four repeats were used for all tests (N = 4).

Next, the molar ratio of H1:H2–4 was optimized, and the results showed that the leakage reaction slowly increased while the concentrations of H2–4 increased. Additionally, 100 nM Fam-H1-BHQ plus 200 nM H2–4 reached the greatest signal-to-noise ratio in the presence of 20 nM initiator I1 (Fig 2A). A molar ratio of H1:H2–4 = 1:2 was applied for the following experiments.

The LOD for I1 determined by the reported HCR system was finally measured. Fig 2B shows that a higher concentration of the hairpins used always resulted in a higher background in the absence of the initiator [1,12]. The signals were barely detected when 25 nM H1 was used. The best LOD was obtained when H1 (100 or 200 nM) was applied. It was concluded that the reaction conditions were optimized as 100 nM H1:200 nM H2–4 and reaction for 30 min at RT, and an LOD for I1 was determined as 2.5 nM under optimized conditions.

DNA/RNA hybridization naturally occurs with high fidelity in organisms within a neutral pH range (pH 6.0–8.0) [39]. DNA/RNA replication is a magnesium-dependent enzymatic process, and magnesium is reported to affect the fidelity of nucleotide hybridization, although it did not show any effects at the tested concentrations. However, according to a previous report, magnesium (12.5 mM) and a reaction at pH 8.0 were applied in the following experiments [12].

After adding the DNA initiator, the fluorescence intensity increased over time because of the programmable hybridization, and it was apparent that a higher concentration of hairpin used resulted in higher background. The addition of extra hairpins H2–H4 also accelerated the hybridization reaction, and most importantly, the reaction rate significantly decreased after 30 min. The best LOD of 2.5 nM for DNA initiator I1 was obtained after 30 min at RT when a molar ratio of H1:H2–4 = 1:2 was used.

The hybridization cascade sped up while the temperature increased; however, it also triggered the unfavored and unspecific hybridization, resulting in a higher background that deteriorated the LOD [2,9,37,39]. Given that heating up the reaction was an energy-consuming step that complicated the test procedures, the reaction was carried out at RT in the following experiments.

Hairpin hybridization is a dynamic equilibrium process, and the addition of reactants drives the reaction towards hybridization. Considering that the labeled H1 is expensive, we decided to increase the amount of the other three hairpins to achieve more hybridization. The results showed that the best LOD was obtained from the tested groups when a molar ratio of H1:H2–4 ≥ 2 was used.

### Selection and specificity of DNA dampeners

A series of dampeners (50 nM) was supplemented in the presence of a DNA initiator (50 nM), and the reaction was carried out using the optimized conditions before fluorescence measurement. The results showed that dampener D5 showed the best inhibitory effect and inhibited greater than 80% of the hybridization caused by the initiator (Fig 2C). To further evaluate the specificity of this method, single-mismatched DNAs were applied and the results indicated that the mismatched DNAs caused 24 to 78% inhibitory of that caused by D5 depending on the location of the mismatches (Fig 2D). This result indicated a relative lower specificity compared to entropy-driven HCR and the specificity of the target was crucial and apparently affected the accuracy of this method.

In the following selection round, D5 was used to inhibit the hybridization caused by the initiator (2.5 nM) that was the LOD of initiator I1. The results showed that D5 significantly inhibited the hybridization caused by I1 (2.5 nM) at the lowest concentrations of 0.156 nM (Fig 2E). However, based on the principle, this method could not be used for quantitative detection but only as a qualitative measure. Qualitative analysis was generally used for screening and early diagnosis of diseases such as COVID-19.

After forming the initiator-H1-H2-H3 complex, the sticky toehold region of H3 (for H4 or dampener binding) was exposed. The dampeners competed with H4 to bind with the exposed sticky strand of H3. H4 is a hairpin with 15 base pairings, and after it hybridized with H3, the base pairings were equal to the previous; however, the dampeners are linear strands, and 20 extra-base pairings were newly formed after hybridizing with H3. Therefore, the H1-H2-H3-H4 hybridization cascade was an entropy-driven process, while dampener incorporation was driven by entropy and enthalpy [37,39–41]. Theoretically, the initiator-H1-H2-H3 was prone to hybridize with the dampeners, which could effectively prohibit the chain hybridization reaction. A longer linear dampener was more energetically favorable. However, D5 is the shortest dampener with the most effective inhibitory effect on the hybridization cascade. This implies that the unspecific interaction caused by the extra nucleotide base had an intensive impact on the DNA pairing in this case.

This study reported that the best LOD for the initiator was 2.5 nM. For most of the reported systems for HCR or CHA, the LOD was reported at the nM scale, which aligned with the current study. In comparison, the LOD for the dampener was 0.156 nM which was 16 times lower than that obtained for the initiator. It was difficult to further improve the LOD for the initiator. However, it would be easier to inhibit the signal caused by the initiator at the current LOD concentration. From the aspect of theoretical energy simulation, we demonstrated that the initiator-H1-H2-H3 was prone to hybridize with the dampeners.

### Polyacrylamide gel electrophoresis

**Fig 3:**
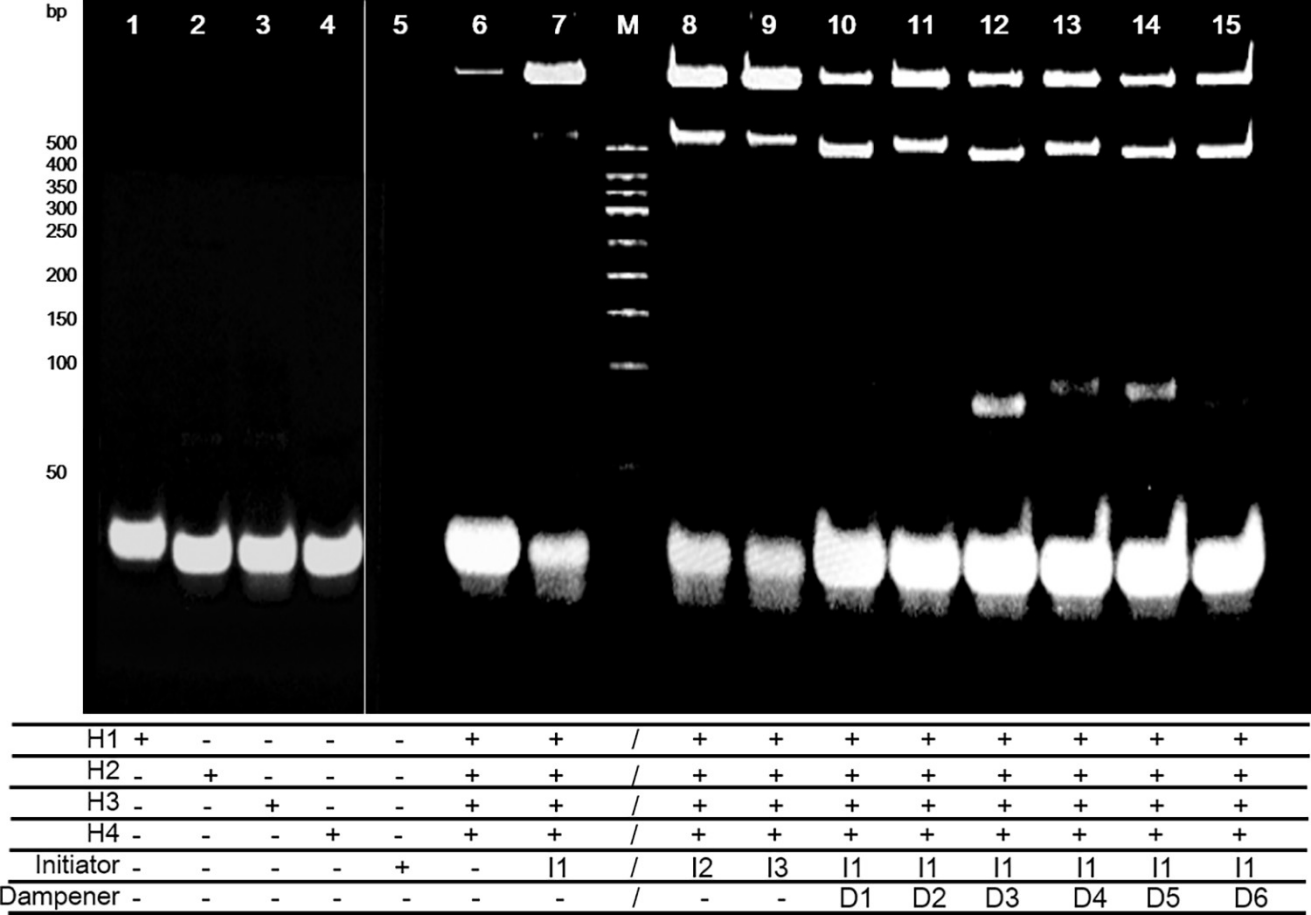
Polyacrylamide gel for the HCR reactions.

The feasibility of this study was illustrated by polyacrylamide gel electrophoresis (Fig 3). The results showed that the initiator I1–3 could trigger the chain hybridization reaction cascade and produce a large complex that was present at the top of the gel well. After adding the dampeners, the HCR cascade was effectively prohibited, and a clear band above 500 bp besides the large complex was observed in all test groups. Some small bands of about 100 bp were also observed in the middle of the gel, illustrating that the dampeners were prone to be incorporated to form a complex of short length.

### HCV-RNA detection

**Fig 4:**
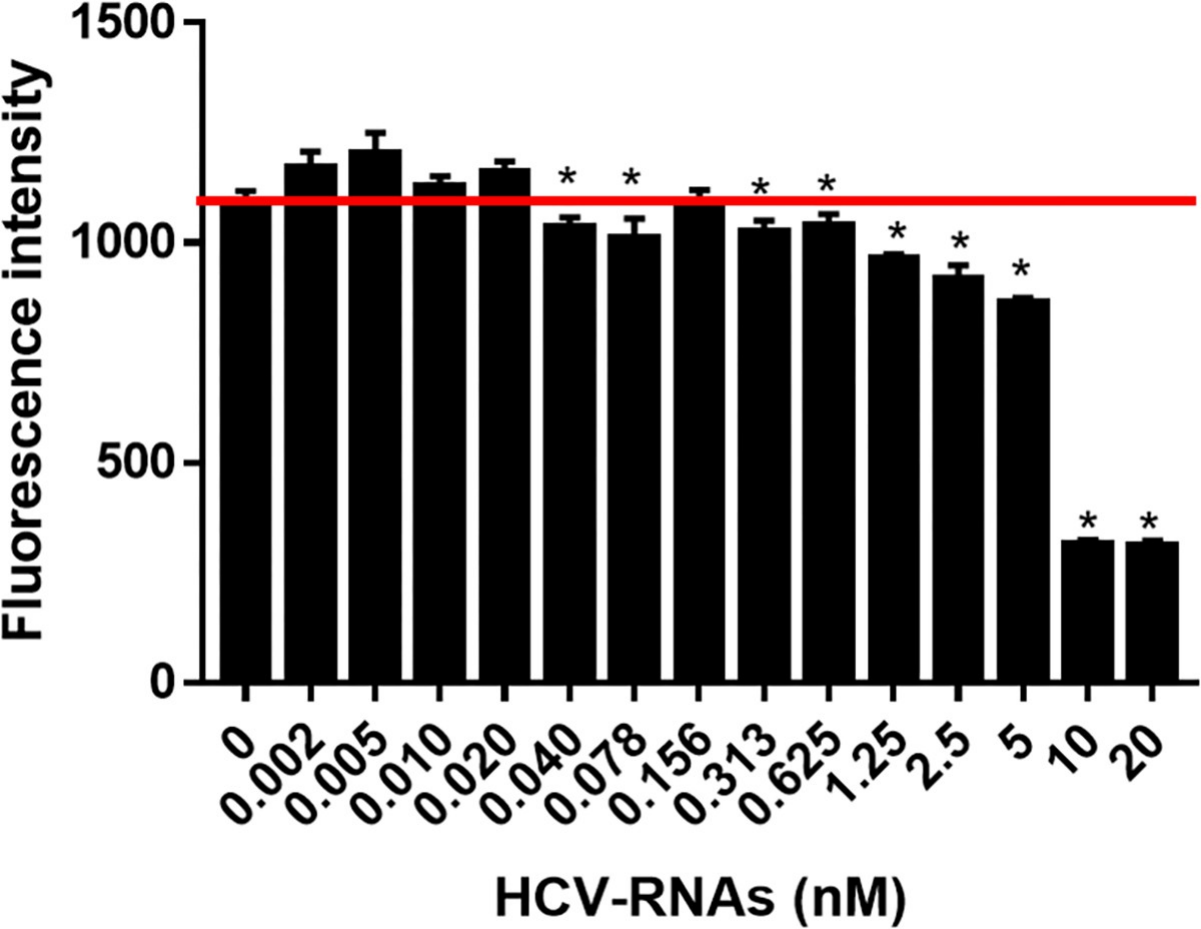
Detection of the HCV-RNAs. * *p*<0.05 vs. blank control, n = 3.

The study results showed that the inhibitory effect was not correlated with the HCV-RNA concentrations (Fig 4). Low concentrations of HCV-RNAs below 0.02 nM caused unspecific leakage in addition to inhibition of the HCR compared with the blank control. At concentrations greater than 0.04 nM, HCV-RNAs showed an inhibitory effect on the HCR except for the 0.156 nM concentration. We also found that there were always inversion results present in the middle of the curve, especially at concentrations lower than the LOD. It was observed that the addition of unrelated DNAs resulted in nonspecific leakage reactions. There might have been a balance between triggering the programmed cascade and incurring unspecific leakage, depending on the concentrations used in the reaction. Referring to the above results, this method’s detection limit for the HCV-RNAs was 0.625 nM.

PCR was generally applied in clinic for sensitive detection of HCV RNA. PCR is a laboratory method requiring for clean bench, precise instruments, and, most important, the professional technicians [43]. As a non-enzymatic isothermal method, hybridization chain reaction (HCR) method was cheap in cost and simple in the test procedures that made it very suitable to be coupled with other signal amplification method for point-of-care testing [12,13]. As a mature and standard detection method, fluorescent detection had be widely applied in clinic with merits such as reliable performance and easy access to the instruments. Thus, fluorescence detection based on HCR was chosen for HCV detection in this study.

## Conclusion

We developed a method similar to competitive ELISA. The HCR reaction conditions have been optimized, and the LOD for the initiator DNA that could trigger the HCR cascade was measured. Based on the optimized conditions, a series of dampeners that could terminate the HCR cascade were designed and used to inhibit the HCR cascade triggered by the initiator at the concentration of its LOD. Using the lowest concentration of dampeners that could prohibit the HCR cascade, this method can qualitatively detect the presence of dampeners that are single-stranded RNA/DNA.

## Supporting information

S1 Fig The results of the simulation for the secondary structure and free energy of the DNAs by NUPACK. (TIF)

S2 Fig The optimization of the initiators. (TIF)

S3 Fig Hairpins hybridization caused by different concentrations of I1 over time. (TIF)

S1 Table The single mismatched DNAs to D5. (DOCX)

S1 File Original data for graphs. (DOCX)

## Abbreviations

HCR: Hybridization chain reaction
ELISA: Enzyme-linked immunosorbent assay
HDMSA: Hairpin DNA-mediated strand assembly
CHA: Catalytic hairpin assembly
LOD: Limit of detection
HCV: Hepatitis C virus
