# Supplementary figures and images for: Detection of Hepatitis C virus RNA using a novel hybridization chain reaction method that competitively dampens cascade amplification
Source: PLoS One. 2023 Mar 10;18(3):e0268917. doi: 10.1371/journal.pone.0268917 (PMC10004832; doi:10.1371/journal.pone.0268917)

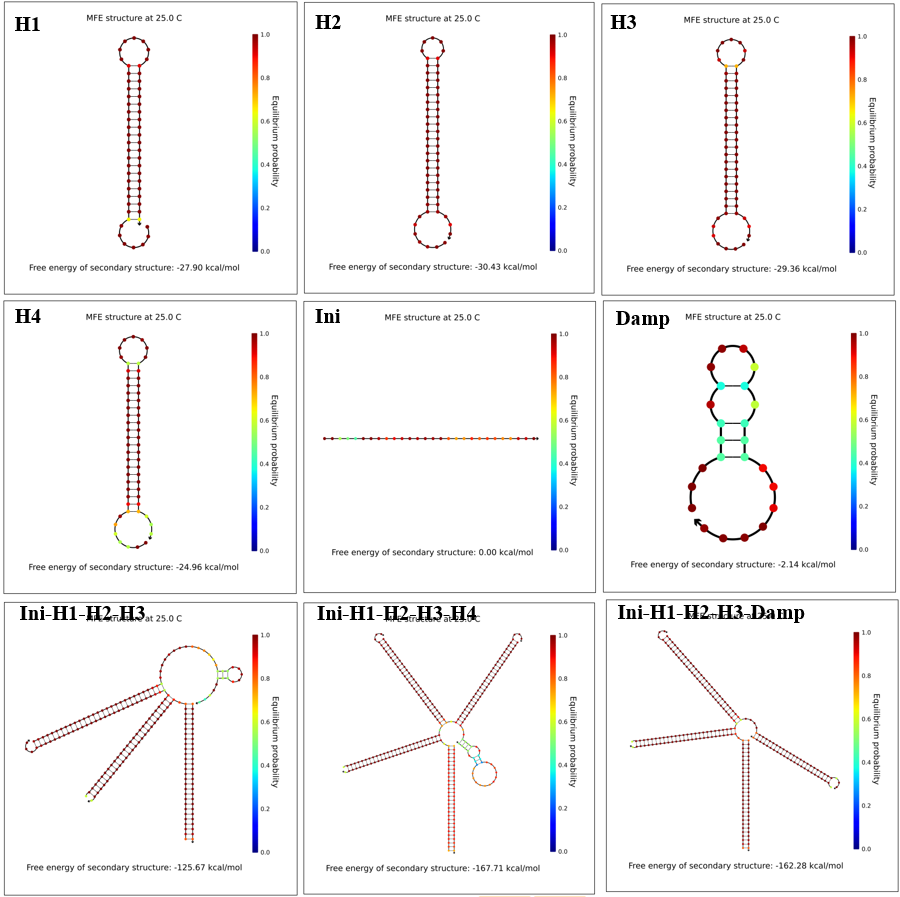

Supplement: S1 Fig — (TIF) [file pone.0268917.s001.tif]

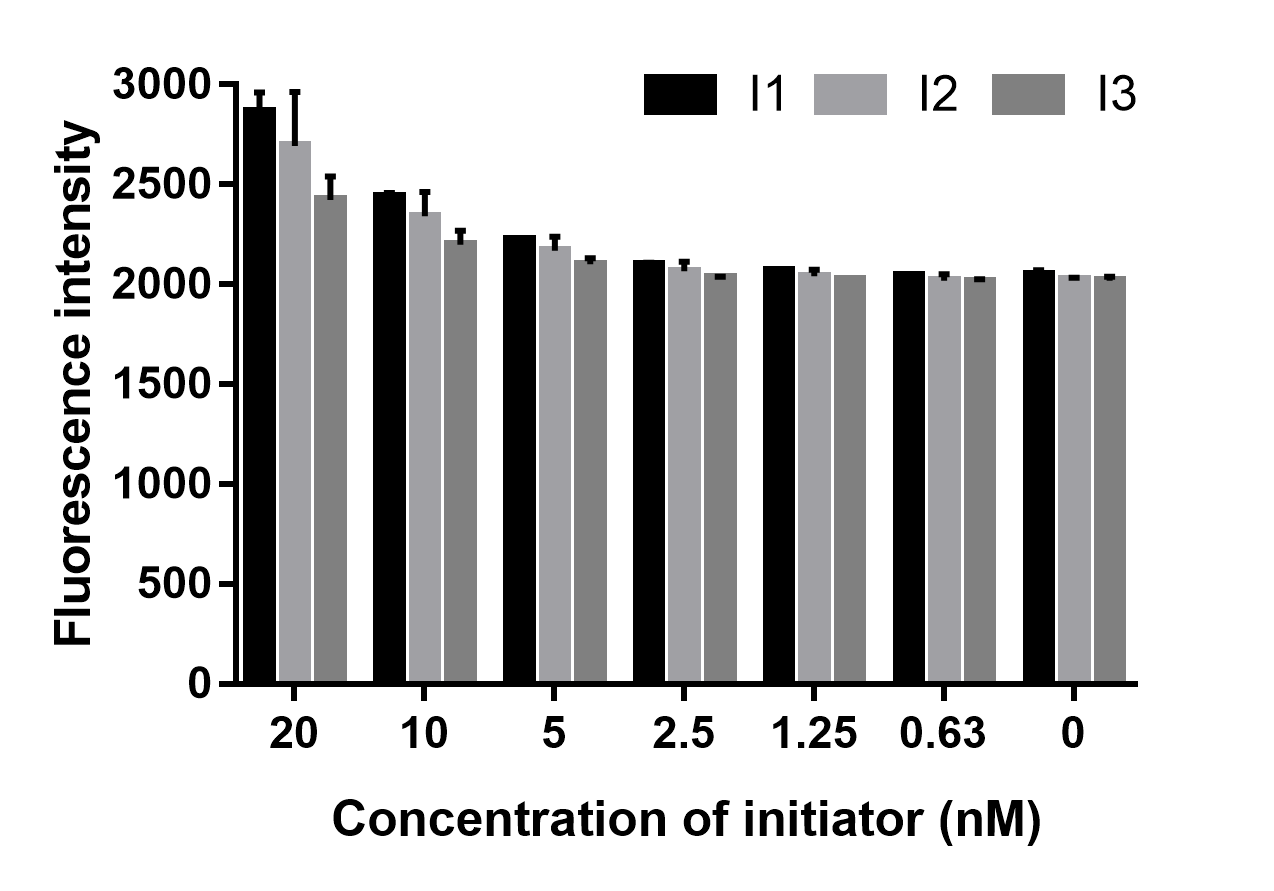

Supplement: S2 Fig — (TIF) [file pone.0268917.s002.tif]

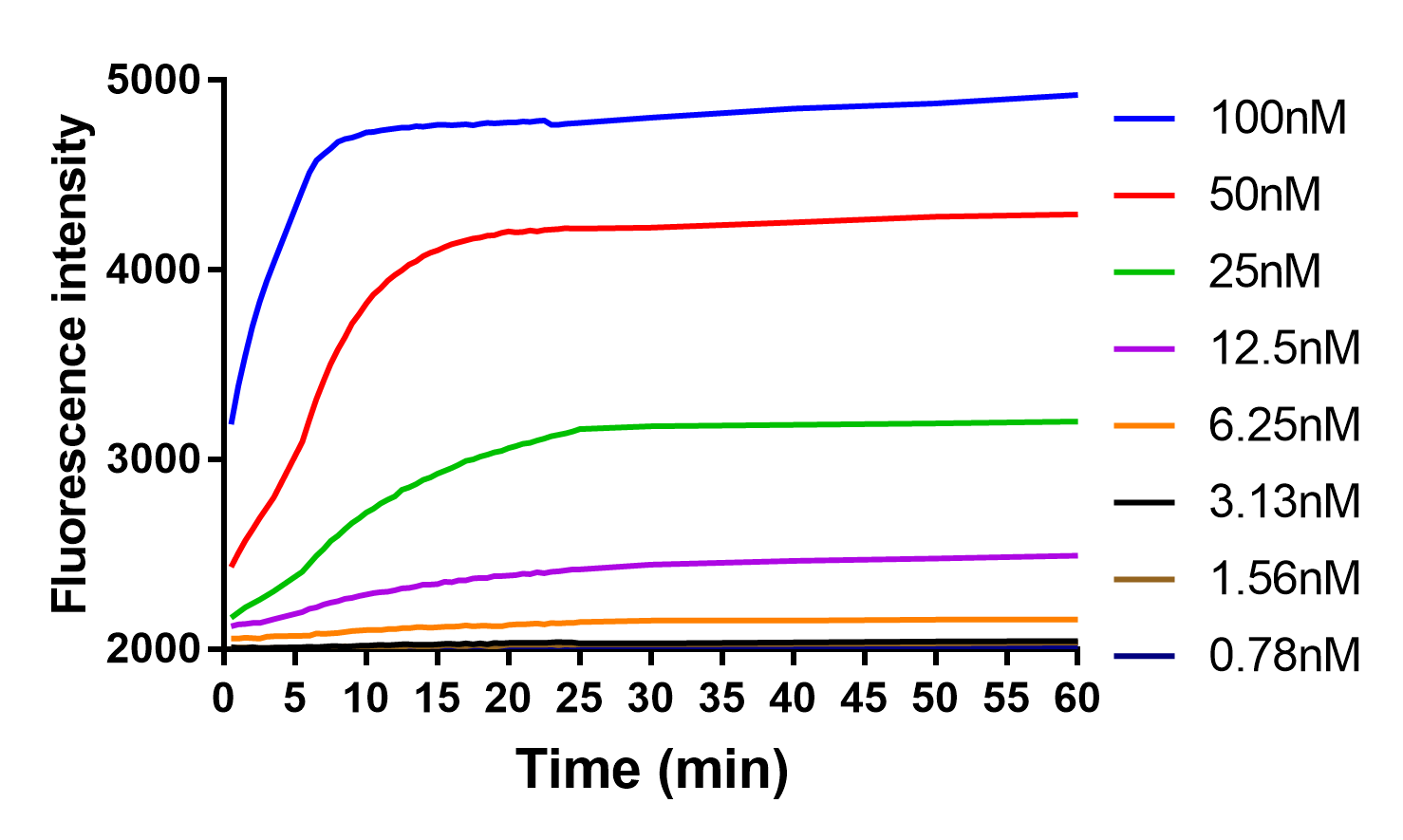

Supplement: S3 Fig — (TIF) [file pone.0268917.s003.tif]
