# Supplementary material for: Detection of Hepatitis C virus RNA using a novel hybridization chain reaction method that competitively dampens cascade amplification
Source: PLoS One. 2023 Mar 10;18(3):e0268917. doi: 10.1371/journal.pone.0268917 (PMC10004832; doi:10.1371/journal.pone.0268917)
Supplement: S1 Table — (DOCX) [file pone.0268917.s004.docx]

**Tables**

**Table S1.** The single mismatched DNAs to D5

| DNA product | Mismatched DNAs |
| --- | --- |
| D5  M1 | **TGGCATGGGATATGATGATGAAGT**  **AGGCATGGGATATGATGATGAAGT** |
| M2 | **TGGCTTGGGATATGATGATGAAGT** |
| M3 | **TGGCATGGGTTATGATGATGAAGT** |
| M4  M5 | **TGGCATGGGATATGTTGATGAAGT**  **TGGCATGGGATATGATGATTAAGT** |
