# Supplementary material for: Detection of Hepatitis C virus RNA using a novel hybridization chain reaction method that competitively dampens cascade amplification
Source: PLoS One. 2023 Mar 10;18(3):e0268917. doi: 10.1371/journal.pone.0268917 (PMC10004832; doi:10.1371/journal.pone.0268917)

# Original data for the graphs

Data for Fig.2A


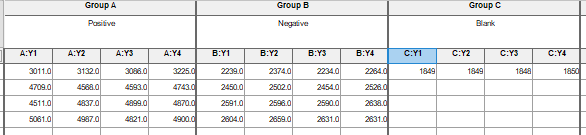


Data for Fig.2B


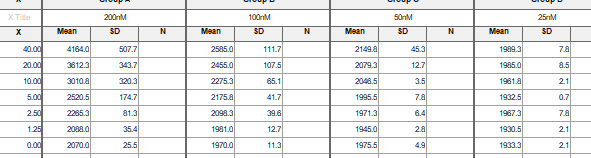


Data for Fig.2C


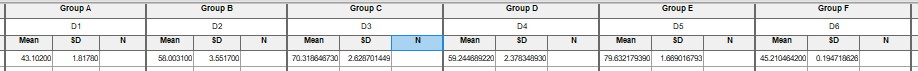


Data for Fig.2D


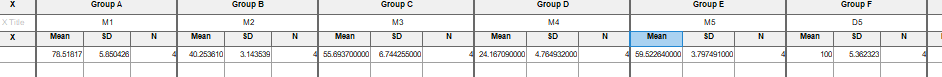


Data for Fig.2E


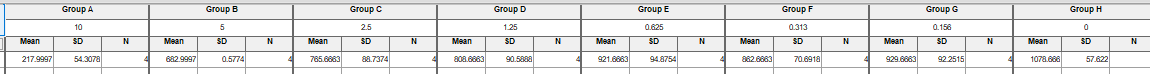


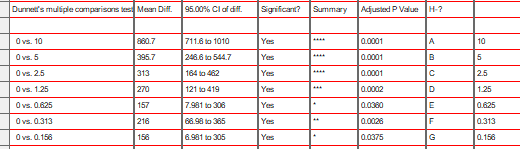


Data for Fig.4


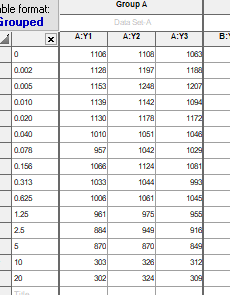

Supplement: S1 File — (DOCX) [file pone.0268917.s005.docx]
